# Formic Acid Stabilization on Supported Ionic Liquid Phases: Insights from Solid‐State NMR Spectroscopy

**DOI:** 10.1002/chem.70950

**Published:** 2026-04-02

**Authors:** Yufei Wu, Yuyan Zhang, Walter Leitner, Alexis Bordet, Thomas Wiegand

**Affiliations:** ^1^ Max Planck Institute For Chemical Energy Conversion Stiftstraße 34–36 Mülheim an der Ruhr Germany; ^2^ Institute of Technical and Macromolecular Chemistry RWTH Aachen University Aachen Germany

**Keywords:** CO_2_ hydrogenation, formic acid, ionic liquid, NMR spectroscopy, supported catalysts

## Abstract

Ionic liquids (ILs) are known to promote the catalytic hydrogenation of CO_2_ to formic acid, in particular by shifting the reaction equilibrium through formic acid stabilization. While a broad range of ILs‐formic acid interactions has been described, proposed, and discussed in pure IL systems, the molecular basis for such stabilizing effects is not yet fully understood. Fundamental insights are desired for practically relevant solid catalysts such as those made from supported ionic liquid phases (SILPs). Herein, we apply solid‐state nuclear magnetic resonance (NMR) spectroscopy to probe interactions between formic acid and four selected catalysts comprising ruthenium nanoparticles (NPs) immobilized on SiO_2_‐based support materials including SILPs (Ru@SILPs). As evidenced by ^1^H transverse relaxation times and the efficiency of ^1^H‐^13^C polarization transfers, a reduction in molecular motion of formic acid is observed upon its impregnation on Ru@SILPs with guanidinium‐ or imidazolium‐based IL‐type molecular modifiers compared to the surfaces with alkyl modifiers or pristine SiO_2_. The NMR spectra reveal spatial proximities between formic acid and the cationic surface modifiers pointing to weak chemical interactions with the cationic modifiers. Our findings provide deeper mechanistic insights into the stabilizing role of SILPs for formic acid, with implications for the synthesis of formic acid from CO_2_ hydrogenation.

## Graphical abstract

Solid‐state NMR spectroscopy reveals insights into interactions between formic acid and molecularly‐modified silica surfaces. Our findings contribute to a mechanistic understanding of the stabilization of formic acid by ionic liquid structures, with significant implications for the formic acid production from CO_2_ hydrogenation and adaptive catalysis using the CO_2_+H_2_⇔HCOOH equilibrium.

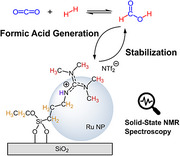

## 1 Introduction

**SCHEME 1:**
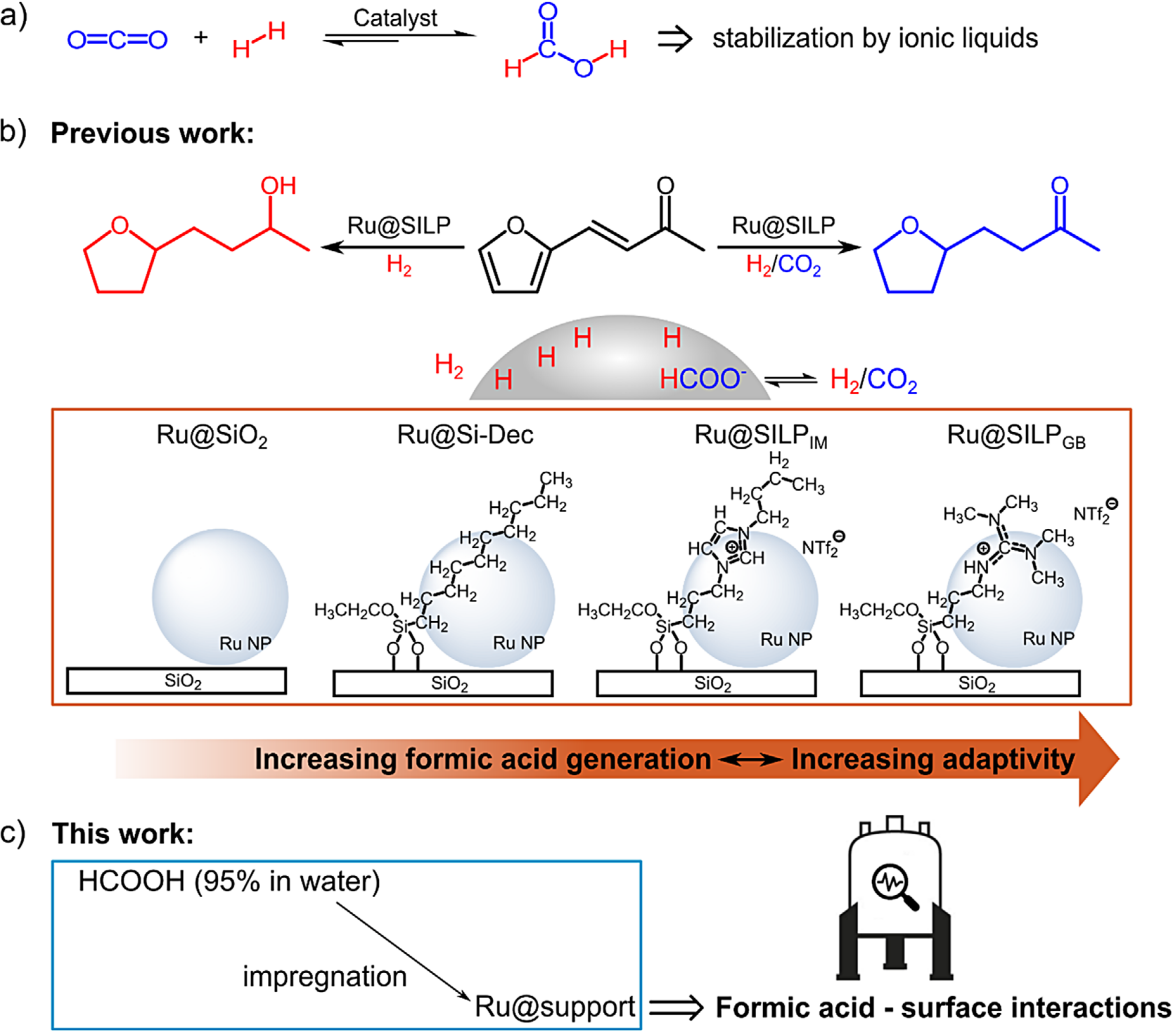
(a) Thermodynamic equilibrium for formic acid formation upon hydrogenation of CO_2_, which is shifted to the formic acid side in presence of ILs. (b) Previous work: CO_2_‐triggered adaptive hydrogenation of ketone‐containing furan derivatives, and an increasing formic acid generation was found for the Ru@SILP catalysts in connection with the increasing hydrogenation selectivity [20]. The chemical structures of the four investigated catalyst materials (Ru@SiO_2_, Ru@Si‐Dec, Ru@SILP_IM_, and Ru@SILP_GB_) are shown, wherein NTf_2_^−^ stands for bistriflimide anion. (c) This work: Probing formic acid stabilization on the modified surfaces sketched in (b) by solid‐state NMR spectroscopy.

The catalytic hydrogenation of CO_2_ to formic acid has garnered particular interest due to existing applications of this molecule as well as future perspectives as C1 feedstock and a potential platform for chemical energy storage [1, 2, 3]. Furthermore, the reversible hydrogenation of CO_2_ to formic acid or formate is recognized as a potentially attractive way to store H_2_ [2, 4]. The formation of formic acid upon hydrogenation of CO_2_ in the gas phase is strongly endergonic and thus poses several challenges [4]. Various approaches to achieve significant concentrations of formic acid have been reported, such as performing the reaction in the presence of basic additives, leading however to the formation of formate ions instead of formic acid [1], as well as thermodynamically stabilizing formed formic acid by suitable reaction media, such as DMSO/H_2_O [5]. Ionic liquids (ILs) are capable of stabilizing formic acid in solution and thus shifting the thermodynamic equilibrium to the formic‐acid side (Scheme 1a) [6, 7]. A broad range of interactions between the IL and formic acid have been proposed to favor this shift, including electrostatic attraction, hydrogen bonding as well as dispersion interactions, involving both, the cation and the anion of the IL [6, 8, 9, 10, 11]. In addition, buffering effects of the ILs significantly affect the equilibrium [12, 13, 14].

Recently, some of us showed that ionic liquid‐modified supports used for the immobilization of ruthenium nanoparticles (NPs) on supported ionic liquid phases (Ru@SILP) are promoting formic acid synthesis kinetically and thermodynamically, leading to enhanced productivity [15, 16]. The same stabilizing effect of the SILP material in the reversible generation of formic acid from H_2_ and CO_2_ was exploited by using CO_2_ as molecular trigger to adaptively control the selectivity of metal NPs in the hydrogenation of multifunctional substrates (Scheme 1b) [17, 18, 19, 20]. In previous work, the stabilizing effect of SILP materials in the H_2_ + CO_2_ ⇔ HCOOH equilibrium was studied by determining the amount of HCOOH formed in solution over a series of supported Ru NPs catalysts (structures shown in Scheme 1b): Ru@SiO_2_ (Ru NPs on pristine SiO_2_), Ru@Si‐Dec (Ru NPs on SiO_2_ functionalized with *n*‐decyl groups), Ru@SILP_IM_ (Ru NPs on an imidazolium‐based SILP) and Ru@SILP_GB_ (Ru NPs on a guanidinium‐based SILP) [20]. Under identical reaction conditions, the formic acid concentrations in the reaction mixture increased following Ru@SiO_2_< Ru@Si‐Dec < Ru@SILP_IM_ < Ru@SILP_GB_. Notably, the modulating effect of CO_2_ during hydrogenation of ketone‐containing furan derivatives correlated directly with this trend, showing no effect for Ru@SiO_2_ and effective shut‐down of the ketone hydrogenation for Ru@SILP_GB_. To obtain a molecular understanding of the stabilizing interactions between formic acid and the SILP materials, we herein employed solid‐state Nuclear Magnetic Resonance (NMR) spectroscopy to directly probe these interactions at the atomic level (Scheme 1c).

Solid‐state NMR under magic‐angle spinning (MAS) conditions has proven to be a powerful tool in the structural investigation of a variety of noncrystalline materials, including molecularly‐modified silica surfaces [21, 22, 23, 24, 25]. For example, isotropic chemical‐shift values or *J*‐coupling constants serve as valuable reporters for probing the chemisorption or the local structural environments of the catalytically‐active center [26, 27, 28, 29, 30]. Two‐dimensional solid‐state NMR experiments have been shown to provide qualitative information on spatial proximities between adsorbed molecules and the surface [31, 32, 33], while quantitative distance measurements can be achieved by Rotational Echo Double Resonance (REDOR)‐based techniques [34]. Those structural restraints can be further used for deriving 3D structures of supported metal complexes with atomic resolution [35, 36]. In addition to structural information, the motional averaging of anisotropic nuclear interactions allows the analysis of molecular motions on the grafted surfaces [37, 38, 39]. Structural and dynamic properties of physisorbed ILs (without a covalent bonding to the silica surface) have also been studied [32, 33]. In that vein, the sensitivity of NMR observables to the local chemical environment and local molecular motion has enabled the study of interactions between physiosorbed or chemisorbed small molecules and the solid surfaces [40, 41, 42, 43]. Previous studies have for instance already pointed to the *in‐situ* generation of formate species in case of a polymer‐grafted silica material [17, 19].

Herein, we demonstrate that solid‐state NMR spectroscopy not only enables the direct detection of formic acid species on catalyst surfaces, but also provides molecular‐level insights into their interaction with molecularly modified surfaces. Using the four Ru@support catalysts previously used in adaptive hydrogenation research (Scheme 1b), we study the structural and dynamic properties of formic acid and surface modifiers contributing to an understanding of the stabilization of formic acid on SILPs.

## 2 Results and Discussion

### 2.1 Ru@Support Catalysts Characterization—Influence of Formic Acid Impregnation

**FIGURE 1:**
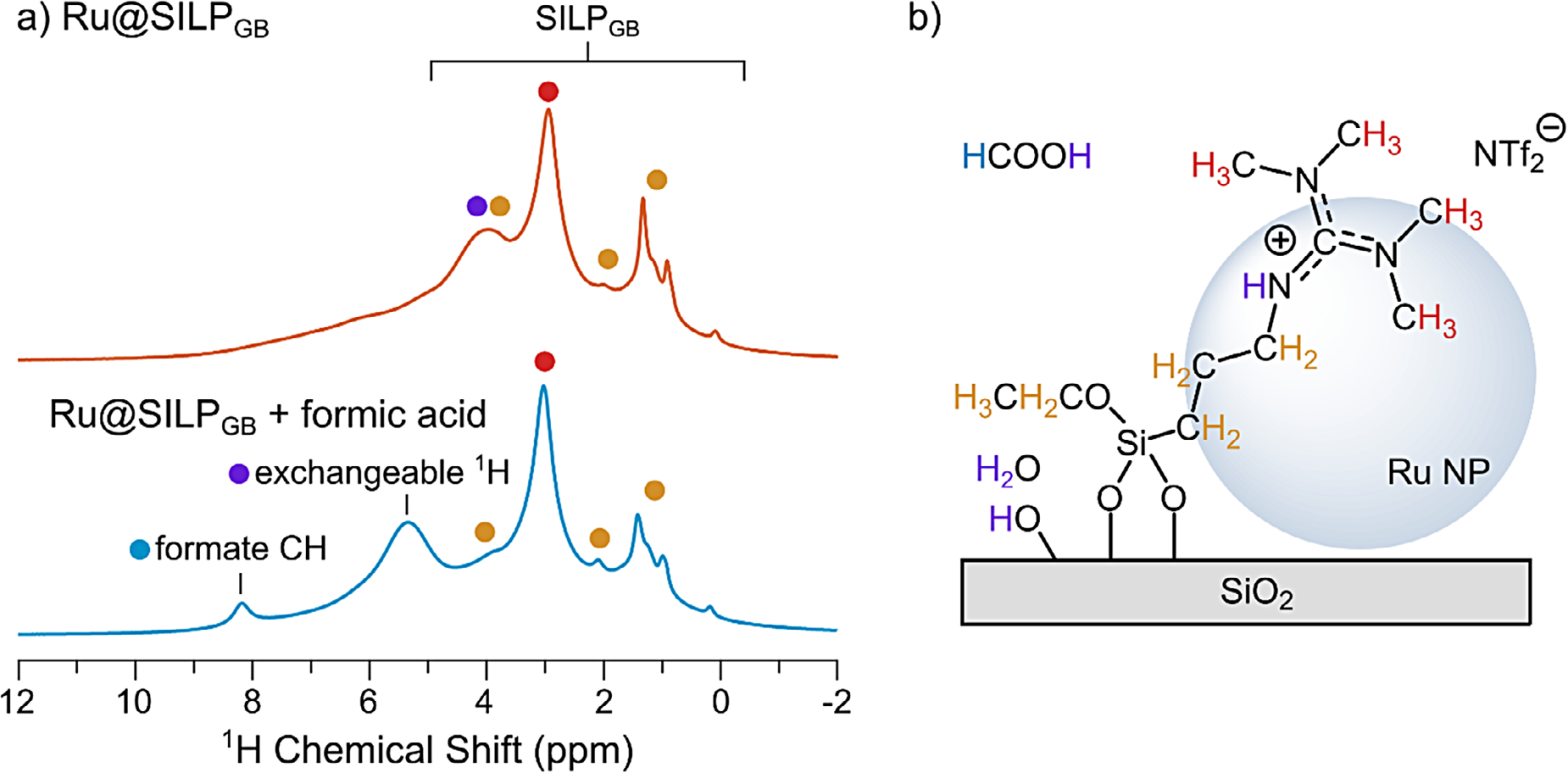
(a) ^1^H solid‐state MAS NMR spin‐echo spectra recorded at a static magnetic‐field strength of 16.4 T and 17.0 kHz MAS of Ru@SILP_GB_ (top) and Ru@SILP_GB_ impregnated with ^13^C‐labelled formic acid (bottom). (b) Schematic chemical structures of Ru@SILP_GB_ and formic acid. The assignment of ^1^H NMR resonances in (a) are indicated by different colors as shown in (b).

The three surface‐modified silica materials (Ru@Si‐Dec, Ru@SILP_IM_, and Ru@SILP_GB_) were characterized by solid‐state NMR spectroscopy to confirm the successful grafting of the molecular modifiers. Several broad resonances are observed in the ^1^H MAS NMR spectra assigned to the molecular modifiers attached to the surface (Figures 1a and S1–S3). ^1^H‐^29^Si Cross‐Polarization (CP‐) MAS NMR spectra reveal resonances in the chemical‐shift range between ‐40 and ‐70 ppm which are assigned to Si atoms connecting the molecular modifiers to the surface (Figures S4–S6), while different ^29^Si chemical‐shift values correspond to different bridging units (denoted as T*_n_* with *n* = 1,2,3 representing the number of C‐Si‐O^surface^ bonds) [27, 44]. For all of the samples investigated not only T_3_ units (‐65 ppm), but also T_2_ (‐57 ppm) and T_1_ (‐48 ppm) units are observed in accordance with previous reports [20]. T_3_ represents complete grafting, T_1_ and T_2_ represent incomplete grafting, where ethoxy groups from the precursors were not completely substituted by the SiO_2_ surface. These residual ethoxy groups also appear in the ^1^H solid‐state spin echo MAS NMR spectra at around 1 ppm (CH_3_) and 4 ppm (CH_2_). The ratios of peak integrals in the ^1^H‐^29^Si CP spectra referring to the different T‐units differ among the samples, with T_2_ dominating for Ru@SILP_GB_ and Ru@Si‐Dec and T_3_ for Ru@SILP_IM_, although such quantifications need to be considered with care, as the CP polarization transfer is *a priori* not quantitative.

In order to mimic the *in‐situ* generation of formic acid as occurring under real catalytic conditions as close as possible for our solid‐state NMR‐spectroscopic investigations, while avoiding using a large quantity of solvent, we have developed a protocol to impregnate the silica materials with formic acid for our spectroscopic studies. In that vein, 100 µL of a 1:49 (v/v) mixture of formic acid (natural abundance or ^13^C isotope‐labelled) and deuterated chloroform (CDCl_3_) have been used to impregnate 40 mg of the Ru@support materials. CDCl_3_ mimics the solvent employed in real catalytic reactions and helps to uniformly distribute formic acid across the entire sample. CDCl_3_ is removed by exposing the impregnated samples to air in a fume hood for 30 min and the solid samples are transferred into the MAS NMR rotors. ^1^H‐^29^Si CP‐MAS NMR spectra confirm that the surface modifications stay intact after impregnation (Figures S4–S6).

The CH ^1^H resonances of formic acid were detected in ^1^H solid‐state MAS NMR spin echo spectra at chemical‐shift values ranging in‐between 8.0 and 8.5 ppm for all the silica samples studied (Figures 1a and S1–S3), which is in agreement with previous reports [17]. In cases where ^13^C‐isotope labelled formic acid was used, these resonances appear as expected as doublets in the absence of ^13^C decoupling during data acquisition which is caused by the one‐bond ^13^C‐^1^H *J*‐coupling (^1^ *J*(^13^C‐^1^H) ≈ 210 Hz, see Figure S7 for Ru@SILP_GB_). A second significantly broadened ^1^H resonance at around 5.5 ppm is assigned to exchangeable protons most likely in (intermediate) chemical exchange involving the ^1^H nuclear spins from the carboxylic acid, physiosorbed water, guanidinium and silanol groups at the surface.

### 2.2 Mobility of Formic Acid on Ru@Supports

**FIGURE 2:**
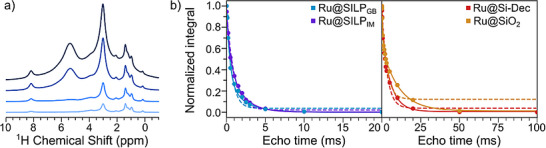
(a) Examples of ^1^H solid‐state spin‐echo MAS NMR spectra of Ru@SILP_GB_ impregnated with formic acid recorded with different spin‐echo times (1.0 µs, 0.2 ms, 1.5 ms, and 3.0 ms from top to bottom). Longer echo delays result in lower intensities. (b) Fitting the spin‐echo decay data to determine *T*_2_ relaxation times. The dashed lines show the least‐square fitting to a single‐exponential decay function and the solid lines show the least‐square fitting to a bi‐exponential decay function (for the fitting parameters see text). Details of the fitting routine are provided in the Supporting Information. The experiments were performed at a magnetic‐field strength of 16.4 T and 17.0 kHz MAS.

Although formic acid is liquid under ambient conditions, the ^1^H resonances of impregnated formic acid that we have identified in the ^1^H MAS NMR spectra are much broader than expected for liquid formic acid, which should appear as sharp resonances due to fast isotropic motion. The line broadening thus points to reduced motion of formic acid on the solid silica surfaces, leading to residual ^1^H‐^1^H homonuclear dipolar interactions causing a homogenous broadening of the ^1^H resonances expressed by a shortening in transverse relaxation times (denoted in the following as *T*_2_ relaxation time) [45, 46, 47]. Therefore, we employ the ^1^H *T*_2_ relaxation times of formic acid as a probe for the molecular mobility and explore whether differences are observed for the different materials. The *T*_2_ relaxation times have been determined by ^1^H spin‐echo decay curves that have been recorded for all impregnated catalysts (see Figures 2a and S8). *T*_2_ relaxation times have been determined from the spin‐echo decay curves by fitting the decay of the integrated formic acid CH ^1^H resonance to exponential decaying functions (Figure 2b, for more details see Supporting Information).

As illustrated by the dashed lines in Figure 2b, single‐exponential fits do not reproduce the decay of the experimental data well for all samples. This is presumably due to a spectral overlap of the formic acid CH resonances with the broad peaks of the exchangeable protons. The latter protons relax faster than formic acid CH and results in a steep decrease at the beginning of the spin‐echo decay curve. This contribution can be correctly captured by fitting the experimental data to a bi‐exponential decaying function (solid lines in Figure 2b), which separates the contribution from fast‐ and slow‐relaxing components. As a result, formic acid impregnated on Ru@SILP_GB_ and Ru@SILP_IM_ showed rather short *T*_2_‐values of 2.0 ± 1.0 ms and 1.7 ± 0.3 ms, respectively. These values are much shorter in comparison to the 11.0 ± 1.0 ms obtained for pristine Ru@SiO_2_, suggesting reduced mobility of formic acid in the presence of IL modifications. For Ru@Si‐Dec, where the surface modifier is a decyl chain, the *T*_2_‐value of formic acid is determined to 5.7 ± 0.6 ms, which ranges in‐between Ru@SILPs and Ru@SiO_2_. The shorter *T*_2_‐values clearly point to a reduced molecular mobility of formic acid when impregnated on molecularly‐modified silica surfaces, with stronger immobilization effects for Ru@SILP_GB_ and Ru@SILP_IM_ with IL‐type molecular modifiers compared to the Ru@Si‐Dec grafted with noncharged decyl chains.

**FIGURE 3:**
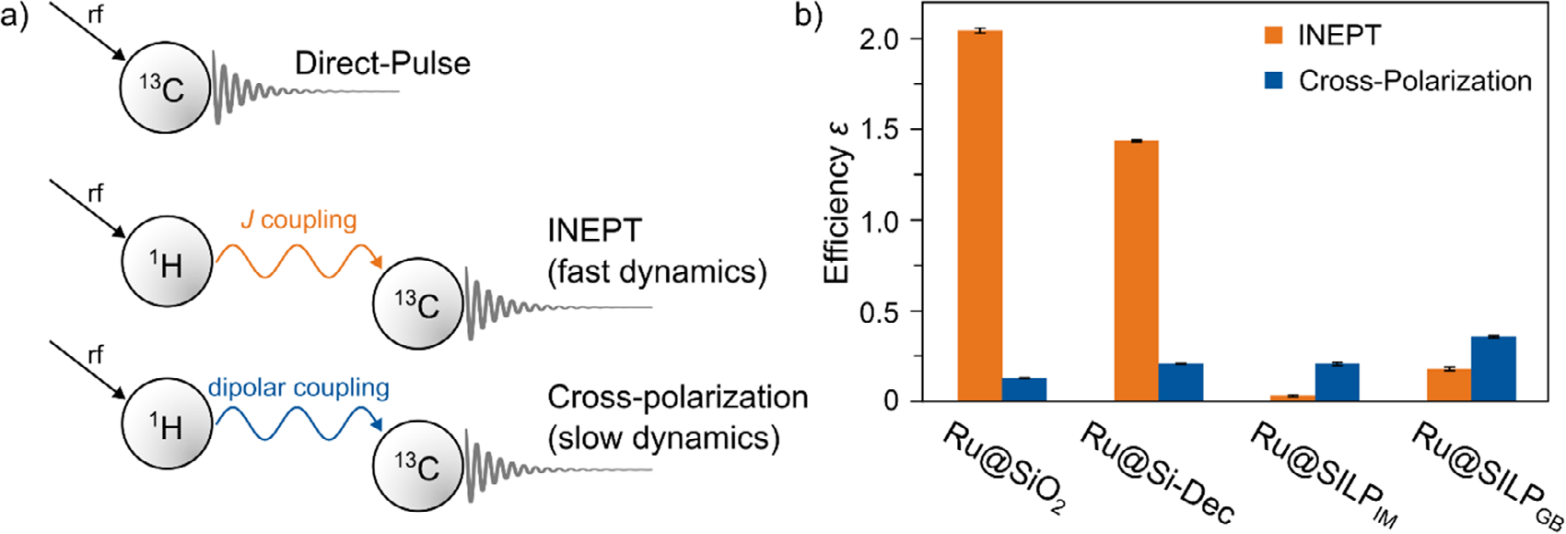
(a) Illustration of the excitation and polarization transfer steps in ^13^C DP, ^1^H‐^13^C INEPT, and ^1^H‐^13^C CP experiments. For the definition of slow and fast dynamics we refer to the main text. (b) Polarization transfer efficiencies (*ε*) of CP and INEPT experiments determined from the ratios between the integrals of the formic acid signal in the INEPT/CP spectra and DP spectra.

The observation of varying immobilization effects of formic acid for the different silica surfaces is further corroborated by comparing ^1^H‐^13^C polarization transfer efficiencies in ^13^C‐detected experiments. ^13^C‐detected solid‐state NMR spectra can be recorded with different experimental techniques, namely Direct‐Pulse (DP), Insensitive Nuclei Enhanced by Polarization Transfer (INEPT), and CP (Figure 3a). In the DP experiment, the ^13^C spectrum is detected directly after excitation, while in the other two experiments, ^1^H spin polarization is transferred to ^13^C nuclei prior to detection. The efficiency of INEPT and CP experiments is dependent on the extent of molecular motions. INEPT relies on the isotropic heteronuclear *J*‐coupling between ^1^H and ^13^C nuclei, and is efficient in case of fast isotropic motions (correlation times below 10^−7^ s). CP in contrast takes typically place via the direct (through‐space) dipole‐dipole interactions between ^1^H and ^13^C nuclei that are close in space, and is most efficient in case of reduced isotropic molecular motions (correlation times longer 10^−5^ s) [48, 49]. Note, that in case of anisotropic motions CP transfer can be observed via the residual heteronuclear dipolar coupling even in case of short correlation times [48, 49]. We have recorded ^13^C spectra using all three techniques. The signal integrals from INEPT and CP spectra are compared to those from DP spectra by determining their respective ratios *ε* as a measure for the polarization transfer efficiencies.

The formic acid ^13^C resonances of all impregnated catalysts resonate at isotropic chemical‐shift values ranging from 164.5 to 166.0 ppm (Figure S9). The efficiencies of the polarization transfer steps are evaluated by calculating the ratio *ε* between the signal integrals of INEPT/CP spectra and DP spectra (Figure 3b). In the absence of ILs, formic acid shows efficient INEPT polarization transfers on both Ru@SiO_2_ and Ru@Si‐Dec (*ε*_INEPT_ = 2.05 ± 0.01 and 1.43 ± 0.01) in contrast to rather inefficient CP polarization transfers (*ε*_CP_ = 0.129 ± 0.003 and 0.210 ± 0.001). This suggests that the impregnated formic acid still undergoes fast isotropic molecular motion on these surfaces. In contrast, in the case of Ru@SILP_GB_ and Ru@SILP_IM_, the INEPT efficiencies decreased to far below one (*ε*_INEPT_ = 0.18 ± 0.01 and 0.03 ± 0.01) indicating a significant reduction of molecular mobility, which agrees with the shorter proton *T*_2_ relaxation times discussed above. There is also an increase in the CP polarization transfer efficiencies (*ε*_CP_ = 0.21 ± 0.01 and 0.36 ± 0.01) compared to Ru@SiO_2_ and Ru@Si‐Dec, but the difference is not as obvious as in the INEPT experiments still indicating some residual motion rendering both, the INEPT as well as CP polarization transfer not very efficient (*vide infra*).

### 2.3 Probing Interactions between Formic Acid and the SILPs

**FIGURE 4:**
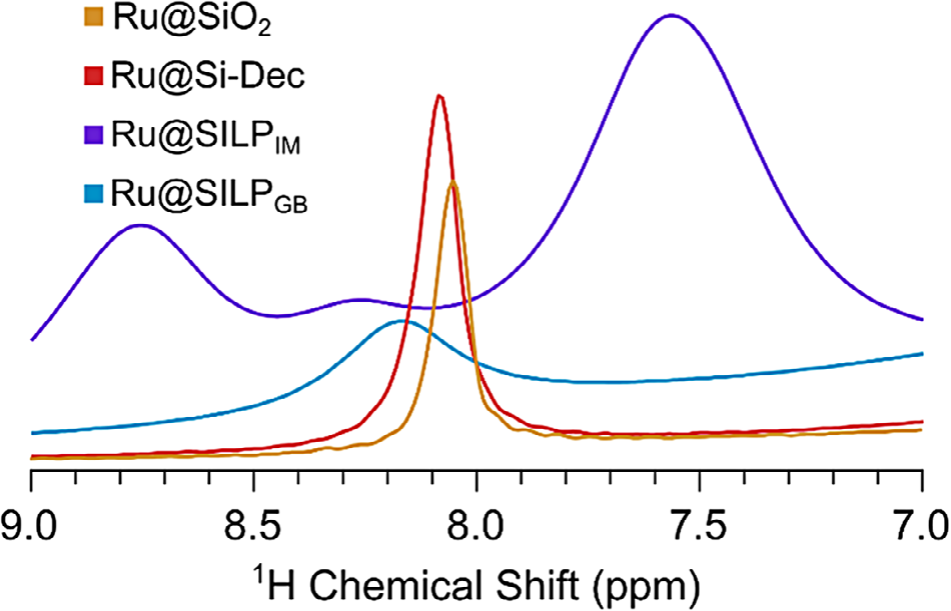
Overlay of zooms into the ^1^H MAS NMR spin‐echo spectra showing the CH formic acid resonance of the impregnated catalysts to illustrate differences in ^1^H chemical‐shift values as well as linewidths, recorded at a magnetic‐field strength of 16.4 T and 17.0 kHz MAS.

We next explored the ^1^H chemical‐shift value of the CH group of formic acid as an indicator for interactions between formic acid and the materials surfaces. The comparison of formic acid CH ^1^H resonances of all four impregnated silica materials are shown in Figure 4 and reveal differences in terms of both, linewidths and isotropic ^1^H chemical‐shift values. The broader linewidths detected for formic acid impregnated on Ru@SILP_GB_ and Ru@SILP_IM_ are in accordance with the shorter proton *T*_2_‐relaxation times caused by reduced mobility reported above. In addition, the ^1^H resonances of formic acid impregnated on Ru@SILP_GB_ and Ru@SILP_IM_ are slightly more deshielded (8.2 and 8.3 ppm, respectively) in comparison to Ru@SiO_2_ and Ru@Si‐Dec (both 8.1 ppm). This observation might indicate an interaction between formic acid and the SILP, although the CH group ^1^H chemical‐shift value of formic acid is influenced by a variety of effects, such as the pH‐value, water concentration, the presence of hydrogen bonds, as well as the protonation state [50, 51, 52, 53].

To probe any close spatial proximity between formic acid and the cationic head group of SILPs, we performed 2D spin diffusion (SD)‐based ^1^H‐^1^H correlation experiments, which reveal correlations between nuclei that are in spatial proximity [54, 55, 56] and are thus frequently explored in structure determination [57, 58, 59]. However, when impregnated on the catalysts, the decomposition of formic acid to CO_2_/H_2_ is catalyzed by the Ru NPs and takes place quickly, which can be observed from the significant decrease of both ^1^H and ^13^C solid‐state NMR resonances after one night (Figure S11). This complicates the acquisition of 2D solid‐state NMR spectra, often requiring several hours of measurement time. Therefore, we decided to perform these experiments on the SILPs without Ru‐NPs, where the decomposition is significantly slower.

**FIGURE 5:**
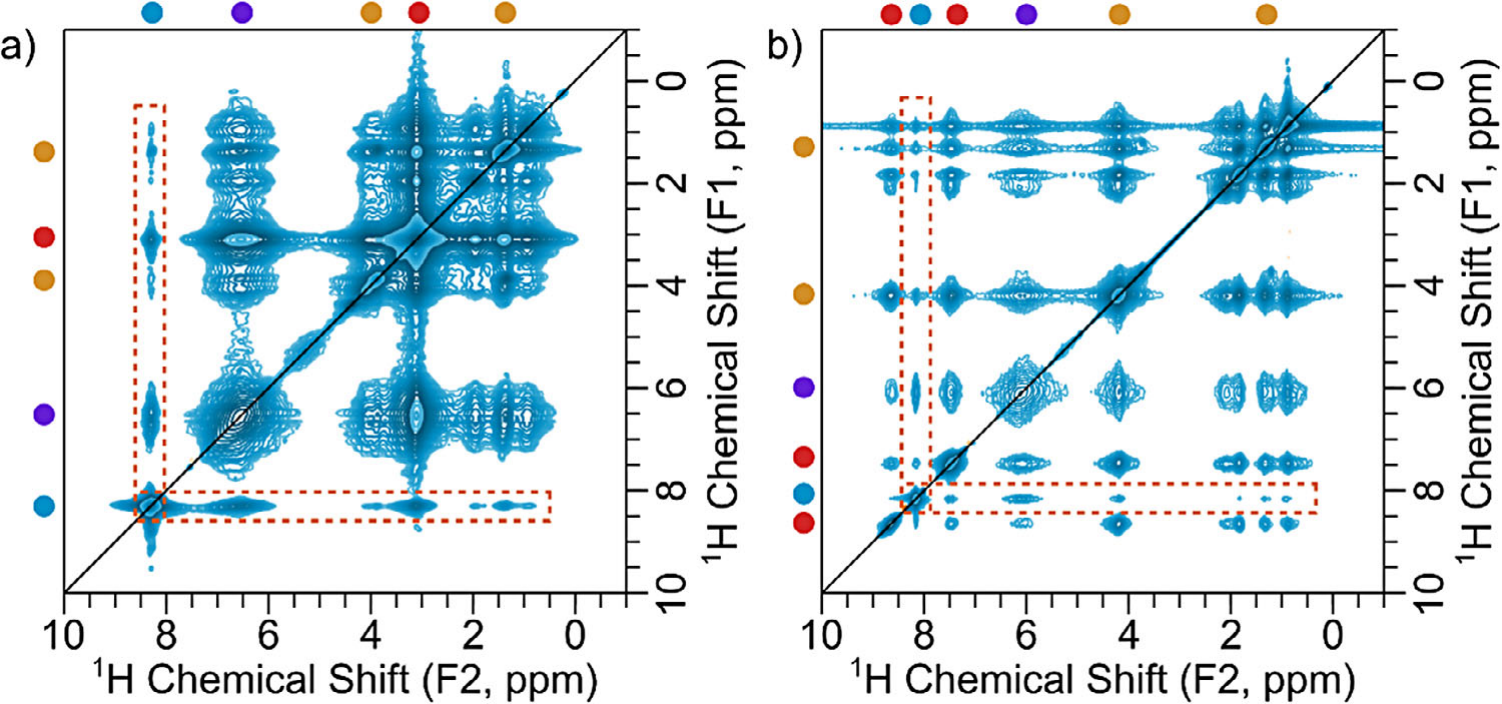
^1^H‐^1^H 2D spin diffusion‐based correlation spectra of (a) SILP_GB_ and (b) SILP_IM_ impregnated with formic acid, both spectra were recorded with 50 ms mixing time at a magnetic‐field strength of 16.4 T and 60.0 kHz MAS.

We used a natural abundance formic acid/CDCl_3_ mixture with a higher formic acid concentration (1:9 v/v) to impregnate the SILPs in order to increase the relative intensity of the formic acid signal. Under this condition, we observed hydrolysis of the SILP but it did not interfere with the measurement (further discussions are included in the Supporting Information and Figure S13). Figure 5 shows the 2D SD‐based ^1^H‐^1^H correlation spectra recorded on SILP_GB_ and SILP_IM_ with a mixing time of 50 ms at 60.0 kHz MAS. The faster MAS frequency used leads to a significant increase in spectral resolution [45, 55, 56, 60]. ^1^H resonance assignments from different chemical environments are labelled with circles in different colors in Figure 5. The cross peaks in the 2D spectra reveal correlations between ^1^H nuclei in close spatial proximity. The correlations with the formic acid CH resonance (^1^H chemical‐shift value at around 8.2 ppm, blue) have been highlighted by orange dashed boxes in Figure 5. Among those, intense correlations to the exchangeable ^1^H nuclear spins at 6.0‐6.5 ppm are observed (purple). In addition, correlations to the cationic IL head groups (red, methyl groups at 3.0 ppm for SILP_GB_ and imidazolium groups at 7.5/8.6 ppm for SILP_IM_) are visible. Weak cross peaks to the aliphatic ^1^H nuclei (1‐2 ppm, yellow) and the ethoxy ^1^H (4 ppm, yellow) are also present. The observation of correlations to all SILP resonances indicates, that the formic acid is not entirely confined to the cationic part of the SILP surface (e.g. by a well‐defined Coulomb‐type interaction with the positively charged IL head groups), but remains to some extent mobile, eventually even interacting with the anion or replacing the latter after deprotonation.^1^ This finding is further supported by ^1^H{^13^C} REDOR experiments [34] performed on the Ru@SILP_GB_ sample using ^13^C isotope labelled formic acid for impregnation. Only a very weak nuclear dipolar dephasing effect less than 2% could be observed for the methyl resonances of the IL head group (Figure S13a). The estimated dipolar coupling strength is only around 20 Hz (Figure S13b), pointing to strong motional averaging effects.^2^ The broad ^1^H resonances prevent the quantitative assessment of the nuclear dipolar coupling constant between the formic acid and further sites on the SILP.

## 3 Conclusion

In conclusion, we have studied interactions between formic acid and a series of SiO_2_‐based support materials loaded with Ru NPs upon impregnating formic acid on such solid supports. ^1^H‐ and ^13^C‐detected solid‐state NMR experiments allowed us to probe the mobility of formic acid species on the surface of such materials. The comparison of ^1^H transverse relaxation times and ^1^H‐^13^C CP/INEPT polarization transfer efficiencies revealed an increase in immobilization of formic acid when it was impregnated on Ru@SILPs with guanidinium‐ or imidazolium‐based ionic liquid structures compared to the surfaces with simple alkyl chains or pristine SiO_2_. In addition, the deshielded ^1^H resonances of formic acid after impregnation on Ru@SILP catalysts and spatial contacts probed in ^1^H‐^1^H SD‐based 2D spectra suggest a weak chemical interaction between the ionic liquid and formic acid. The data clearly reveal a molecular interaction of formic acid with the cationic molecular modifiers, corroborating with the various degrees of stabilizing formic acid and shifting the formic acid formation equilibrium from CO_2_ and H_2_ to the product site. The interactions may result from electrostatic attraction, hydrogen bonding, as well as anion exchange, or even a combination thereof. Formic acid, however, still possesses some motional degree of freedom on the surfaces. The increase in formic acid stability can be correlated with the improved capability of Ru@SILP catalysts to generate formic acid from CO_2_‐hydrogenation and to initiate CO_2_‐triggered reaction selectivity switches. The reported solid‐state NMR approach enables the investigation of interactions between small molecules and molecularly‐modified surfaces. In the future, we will explore solid‐state NMR to further unravel the structural and conformational properties, as well as the dynamics of ILs grafted on the silica surface, focusing also on the detailed interplay with the immobilized NPs essential for catalysis.

## Conflicts of Interest

The authors declare no conflicts of interest.

## Supporting information



The authors have cited additional references within the Supporting Information [61, 62, 63, 64, 65, 66, 67, 68, 69, 70, 71].

## End Notes

1 A potential substitution of the anion by formate is corroborated by washing the SILP with a formic acid solution. The ^19^F solution‐state NMR spectrum of the eluted solution implies a release of the NTf_2_^−^ anion (see Figure S12).

2 The theoretically accessible ^13^C‐^1^H heteronuclear dipolar coupling is additionally reduced by the methyl‐group rotation.
