# Supplementary material for: Formic Acid Stabilization on Supported Ionic Liquid Phases: Insights from Solid‐State NMR Spectroscopy
Source: Chemistry. 2026 Apr 2;32(23):e70950. doi: 10.1002/chem.70950 (PMC13282913; doi:10.1002/chem.70950)
Supplement: Supplementary file 1 — The authors have cited additional references within the Supporting Information [61, 62, 63, 64, 65, 66, 67, 68, 69, 70, 71]. [file CHEM-32-e70950-s001.docx]

Supporting Information for

Formic Acid Stabilization on Supported Ionic Liquid Phases: Insights from Solid-State NMR Spectroscopy

Yufei Wu^+^,^[a,b]^ Yuyan Zhang^+^,^[a]^ Walter Leitner,*^[a,b]^ Alexis Bordet*^[a]^ and Thomas Wiegand^*[a,b]^

[a] Y. Wu,^+^ Dr. Y. Zhang,^+^ Prof. Dr. W. Leitner, Dr. A. Bordet, Prof. Dr. T. Wiegand
Max Planck Institute for Chemical Energy Conversion
Stiftstraße 34-36, 45470 Mülheim/Ruhr (Germany)

[b] Y. Wu,^+^ Prof. Dr. W. Leitner, Prof. Dr. T. Wiegand
Institute of Technical and Macromolecular Chemistry
RWTH Aachen University
Worringerweg 2, 52074 Aachen (Germany)

[^+^] These authors contributed equally to this work.

* Corresponding authors: walter.leitner@cec.mpg.de, alexis.bordet@cec.mpg.de, thomas.wiegand@cec.mpg.de

Chemicals

The procedures and chemicals used for the preparation of the SiO_2_ and molecularly modified SiO_2_ with and without Ru NPs were described in a previous publication.^[1]^

Natural abundance formic acid was purchased from Sigma Aldrich (≥ 95%). ^13^C-labelled formic acid was purchased from Sigma Aldrich (95 wt % in H_2_O, 99 atom % ^13^C). Deuterated chloroform (CDCl_3_) was purchased from Eurisotop (99.80 % D). These chemicals were used without further purification.

NMR spectroscopy

^1^H and ^13^C solid-state NMR spectra were recorded on a Bruker wide-bore 700 MHz magnet (16.4 T magnetic field, ^1^H Larmor frequency of 700.3 MHz, ^13^C Larmor frequency of 176.1 MHz). The lengths of the 90° excitation pulses are 2.5 μs for ^1^H and 5.0 μs for ^13^C corresponding to nutation frequencies of 100 and 50 kHz, respectively. Pulse parameters were optimized on the sample. Recycle delays of the 1D experiments were set to at least 5 times the longitudinal relaxation time to allow quantitative acquisition.  ^1^H and ^13^C chemical shifts were referenced indirectly to tetramethylsilane using adamantane as a secondary standard for ^13^C (**C**H_2_ *δ* = 38.56 ppm).^[2,3]^ The temperature inside the stator was maintained to 280 K leading to a sample temperature of around 300 K.

The ^1^H solid-state spin echo spectra of the catalysts were recorded in 3.2 mm zirconia rotors with a 3.2 mm Bruker double-resonance probe using 17.0 kHz MAS. The echo delay time was set to one rotor period. Unless otherwise noted, heteronuclear decoupling using the WALTZ-16 sequence with 5 kHz rf-field was applied on ^13^C during data acquisition for 1D experiments and during both the echo delay time and acquisition for *T*_2_-measurements.^[4]^

The 1D ^13^C-detected experiments were recorded in 3.2 mm zirconia rotors with a 3.2 mm Bruker double-resonance probe at 17.0 kHz MAS. For the CP-MAS experiments, adiabatic polarization transfer under the Hartmann-Hahn condition was achieved by a ramped-amplitude CP pulse, with *v*_RF_(^1^H) being swept from 48 to 72 kHz using a tangent ramp,^[5,6]^ while the *v*_RF_(^13^C) and CP contact time that gave the best signal intensities were used. Heteronuclear decoupling using the WALTZ-16 sequence with 5 kHz rf-field was applied on ^1^H during data acquisition.^[4]^

The ^1^H solid-state spin echo spectra and the ^1^H-^1^H spin diffusion-based correlation spectra of the impregnated Ru NPs-free materials were acquired in 1.3 mm zirconia rotors with a 1.3 mm Bruker double-resonance probe at 60 kHz MAS. The temperature inside the stator was maintained to 255 K to achieve a sample temperature of around 300 K. The recycle delay of the spin diffusion experiments was set to 1.3 times the ^1^H longitudinal relaxation time.

The ^1^H{^13^C} REDOR experiment were recorded in 3.2 mm zirconia rotors with a 3.2 mm Bruker double-resonance probe at 17.0 kHz MAS. To obtain the reference signal (*S*_0_), the ^1^H 90° excitation was followed by a spin echo with a 5.0 μs 180° ^1^H refocusing pulse in the middle, allowing a total echo time from 0.12 ms to 7.18 ms. To obtain the dephasing signal (*S*’), rotor-synchronized 180° ^13^C pulses with the length of 10.0 μs were applied during the echo delay. The REDOR signal was taken as the relative dephasing ((*S*_0_-*S*’)/*S*­_0_) and plotted against the dephasing time. The simulated curves with nuclear dipolar coupling strength *d* = 10, 20, 30 Hz were calculated with the analytical expression published by Mueller.^[7]^

^1^H-^29^Si solid-state CP-MAS spectra were recorded on a wide-bore Bruker 500 MHz (11.7 T) spectrometer (^29^Si Larmor frequency of 99.4 MHz) with a Bruker 3.2 mm triple-resonance probe at an MAS frequency of 17.0 kHz. The temperature of the cooling gas was maintained at a temperature of 295 K. A ^29^Si-labelled octakis(trimethylsiloxy)silsesquioxane standard sample was used to optimize the ^1^H and ^29^Si nutation frequencies and to reference the ^29^Si chemical shifts (O**Si**(CH_3_)_3_ *δ* = 11.5 ppm relative to tetramethylsilane).^[8]^ After the 2.5 μs 90° excitation pulse for ^1^H, adiabatic polarization transfer under the Hartmann-Hahn condition was achieved by a ramped-amplitude CP pulse, with *v*_RF_(^1^H) being swept from 48 to 72 kHz using a tangent ramp, while *v*_RF_(^29^Si) was kept at 43 kHz. A CP contact time of 5.0 ms was used. 90 kHz SPINAL-64 ^1^H decoupling was applied during data acquisition.^[9]^ Recycle delays were set to three times the longitudinal relaxation time of ^1^H.

Solution-state ^1^H and ^13^C NMR spectra of formic acid were recorded on either a Bruker 400 MHz or a Bruker Ascend 600 MHz spectrometer at room temperature. 30° excitation pulses with the length of 3.33 μs and 3.25 μs were used in 1D experiments for ^1^H and ^13^C, respectively. 90° excitation pulses with the length of 10.0 μs and 9.75 μs for ^1^H and ^13^C were used in the 2D HMBC experiment. The chemical shifts are referenced to tetramethylsilane at 25 °C using the solvent DMSO-d^6^ (*δ*(^1^H) = 2.50 ppm, *δ*(^13^C) = 39.53 ppm) as a secondary reference.^[10]^ Solution-state ^19^F NMR spectra were recorded on a Bruker 300 MHz spectrometer at room temperature. Excitation pulse length was 8.5 μs.

All NMR spectra were processed with the software TopSpin version 4.1.4 and 4.5.0 (Bruker Biospin). The line-shape simulation for spectral decomposition analysis was performed with the software DMFit (release #20230120).^[11]^ 2D spectra were plotted with the software CcpNmr Analysis (version 2.5.2).^[12]^ All spectra are accessible under https://doi.org/10.22000/2tum6jb4hfwpe1rw.

Supplementary Figures


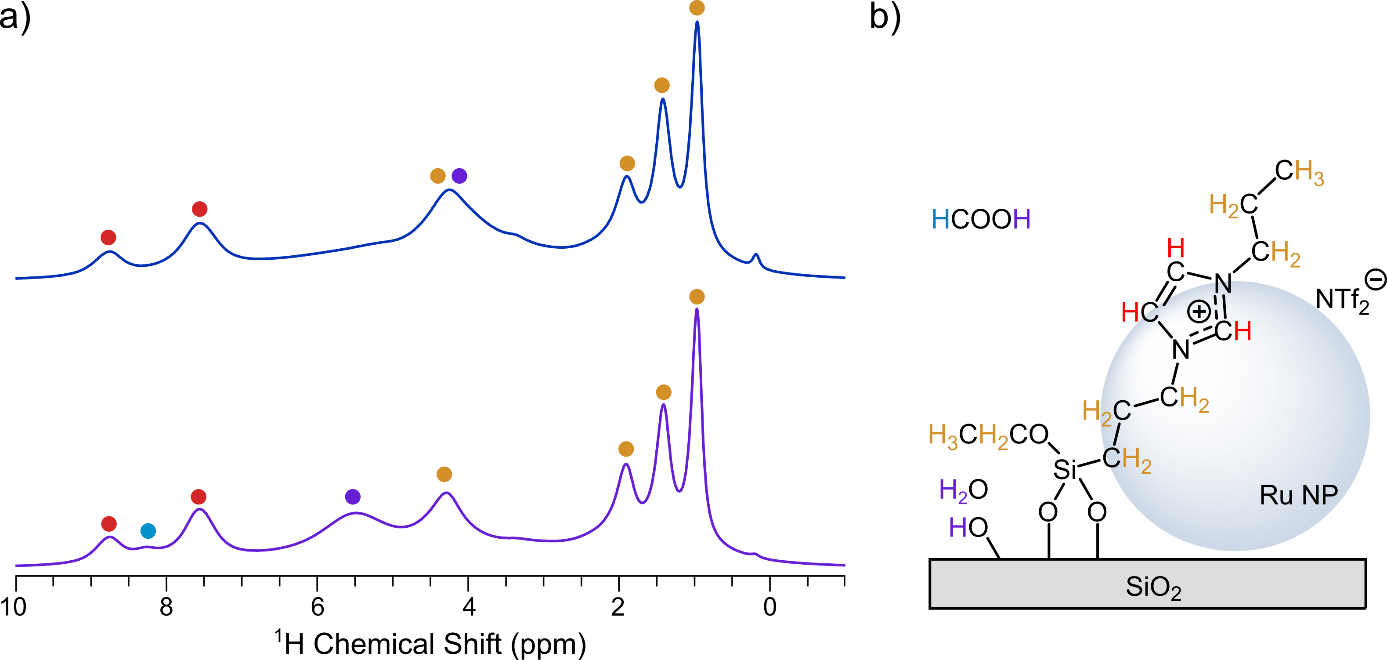


**Figure S1.** ^1^H solid-state MAS NMR spin-echo spectra of Ru@SILP_IM_ (top) and Ru@SILP_IM_ impregnated with ^13^C-labelled formic acid (bottom), recorded at 16.4 T, 17.0 kHz MAS. (b) Schematic chemical structures of Ru@SILP_IM_ and formic acid. The assignment of ^1^H NMR resonances in (a) are indicated by different colors as shown in (b).


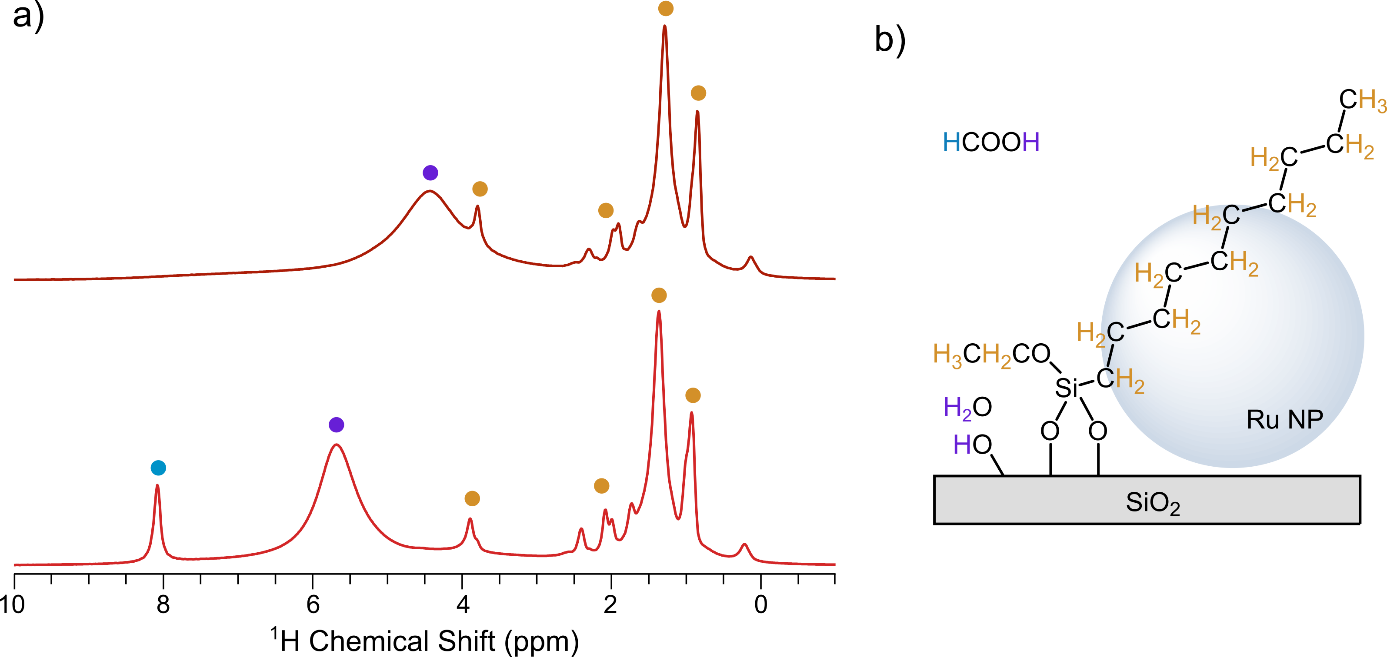


**Figure S2.** ^1^H solid-state MAS NMR spin-echo spectra of Ru@Si-Dec (top) and Ru@Si-Dec impregnated with ^13^C-labelled formic acid (bottom), recorded at 16.4 T, 17.0 kHz MAS. (b) Schematic chemical structures of Ru@Si-Dec and formic acid. The assignment of ^1^H NMR resonances in (a) are indicated by different colors as shown in (b).


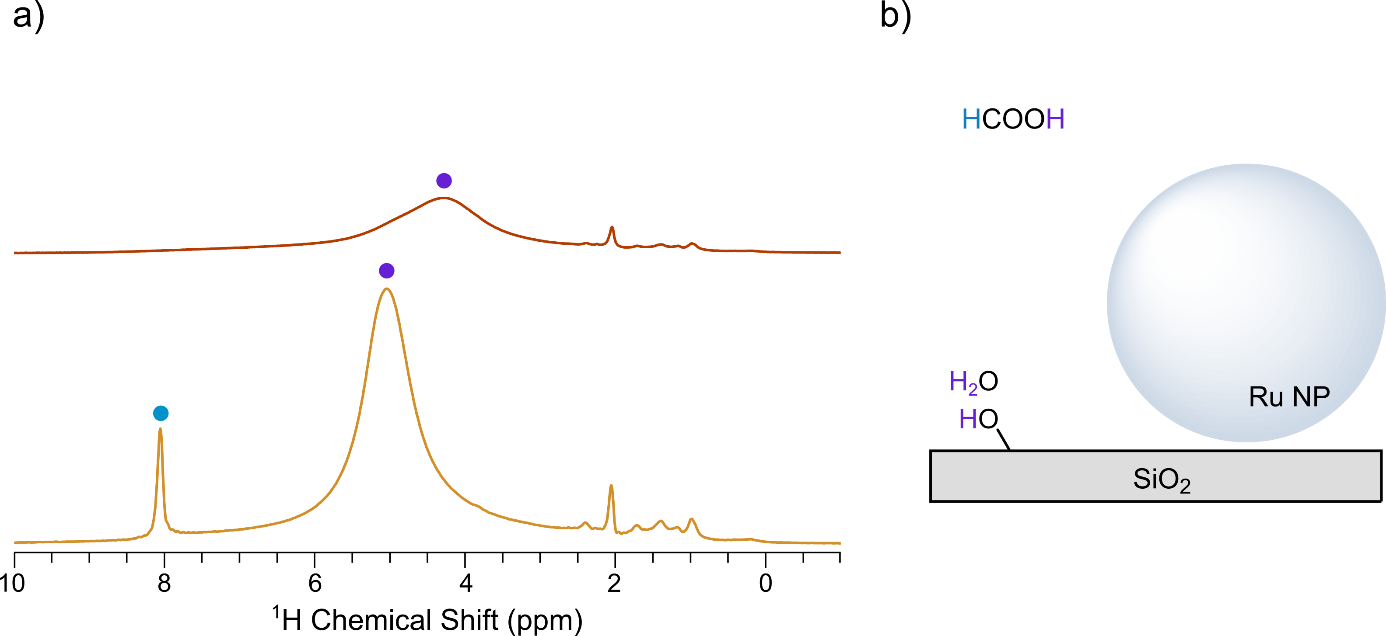


**Figure S3.** ^1^H solid-state MAS NMR spin-echo spectra of Ru@SiO_2_ (top) and Ru@SiO_2_ impregnated with ^13^C-labelled formic acid (bottom), recorded at 16.4 T, 17.0 kHz MAS. (b) Schematic chemical structures of Ru@SiO_2_ and formic acid. The assignment of ^1^H NMR resonances in (a) are indicated by different colors as shown in (b).


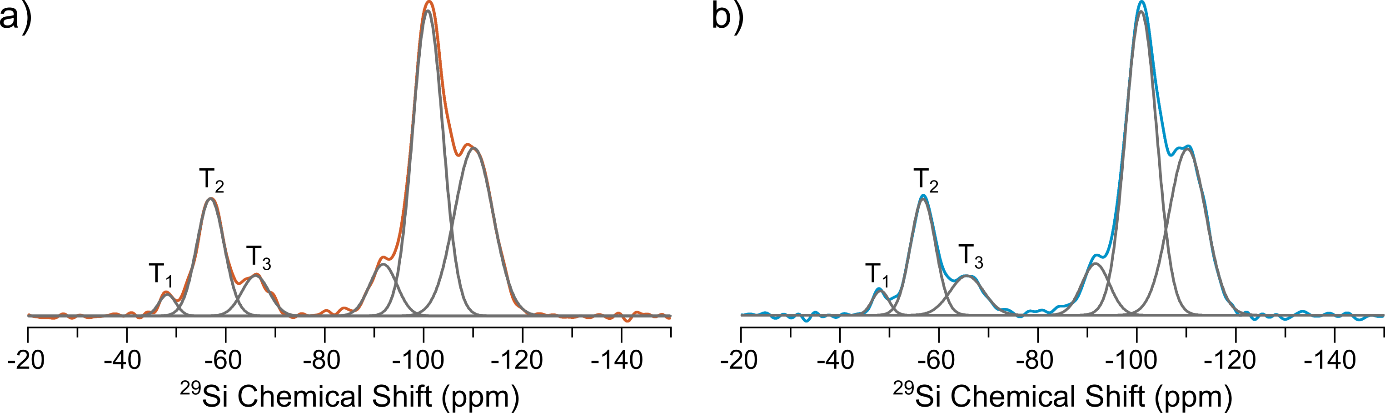


**Figure S4.** ^1^H-^29^Si solid-state CP-MAS NMR spectra of (a) Ru@SILP_GB_ and (b) Ru@SILP_GB_ impregnated with ^13^C-labelled formic acid, recorded at 11.7 T, 17.0 kHz MAS. The lineshape decomposition is shown as well.


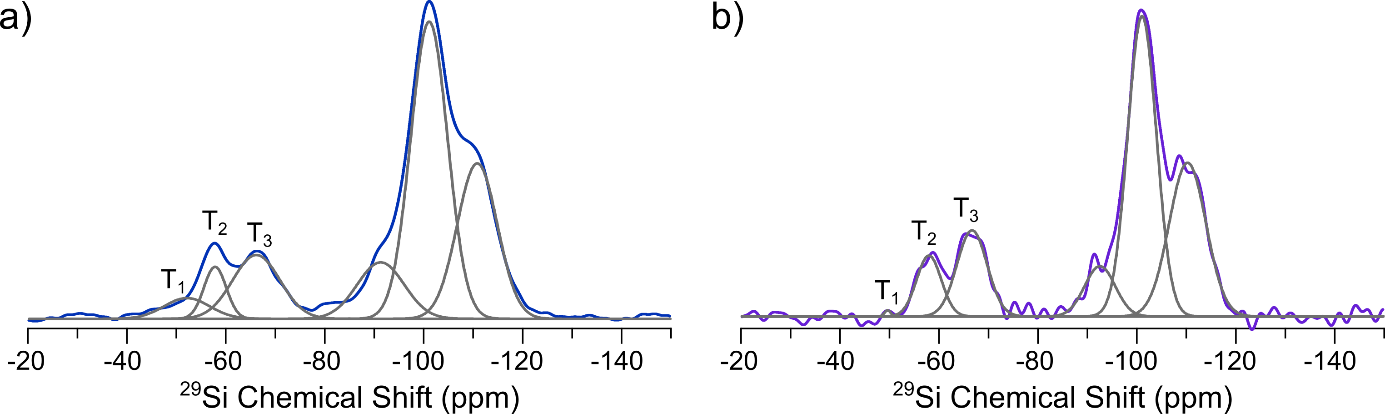


**Figure S5**. ^1^H-^29^Si solid-state CP-MAS NMR spectra of (a) Ru@SILP_IM_ and (b) Ru@SILP_IM_ impregnated with ^13^C-labelled formic acid, recorded at 11.7 T, 17.0 kHz MAS. The lineshape decomposition is shown as well.


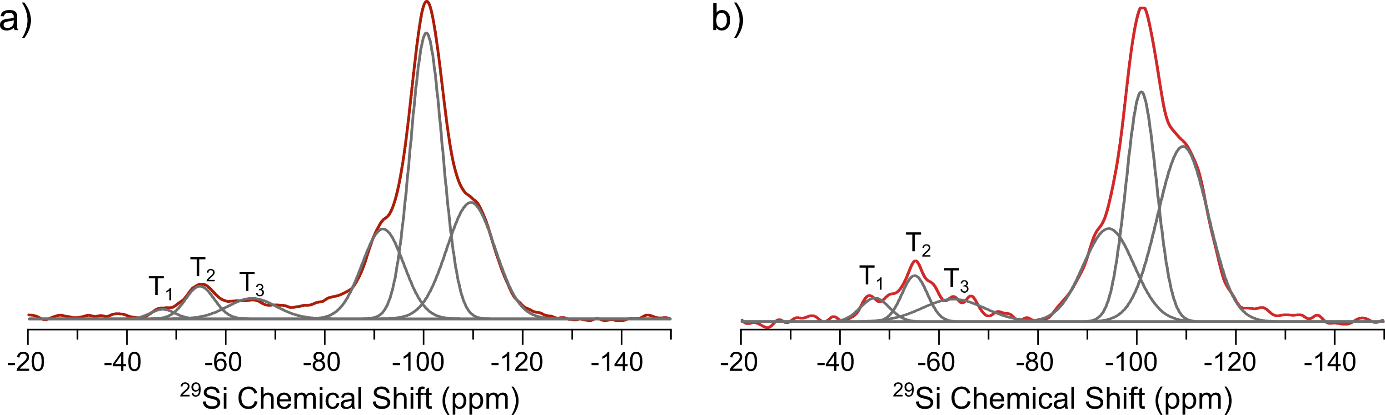


**Figure S6.** ^1^H-^29^Si solid-state CP-MAS NMR spectra of (a) Ru@Si-Dec and (b) Ru@Si-Dec impregnated with ^13^C-labelled formic acid, recorded at 11.7 T, 17.0 kHz MAS. The lineshape decomposition is shown as well.


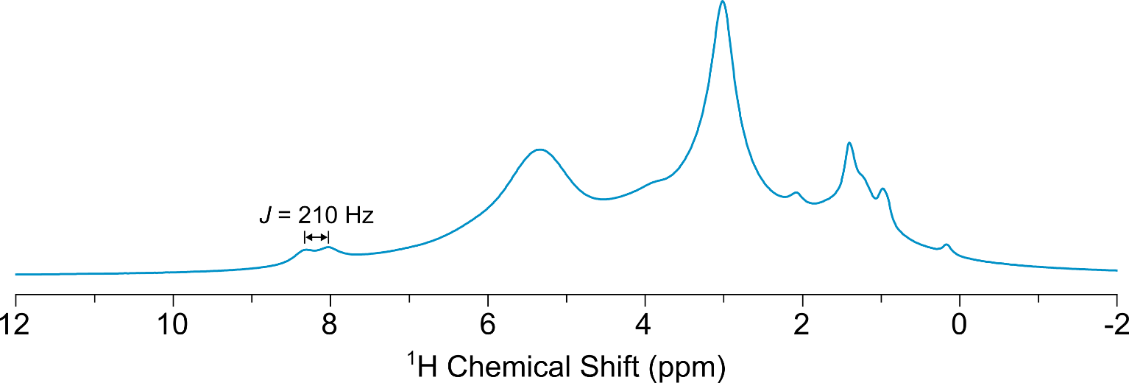


**Figure S7.** ^1^H solid-state MAS NMR spin-echo spectrum of Ru@SILP_GB_ impregnated with ^13^C-labelled formic acid recorded without heteronuclear ^13^C decoupling during data acquisition at 16.4 T and 17.0 kHz MAS.


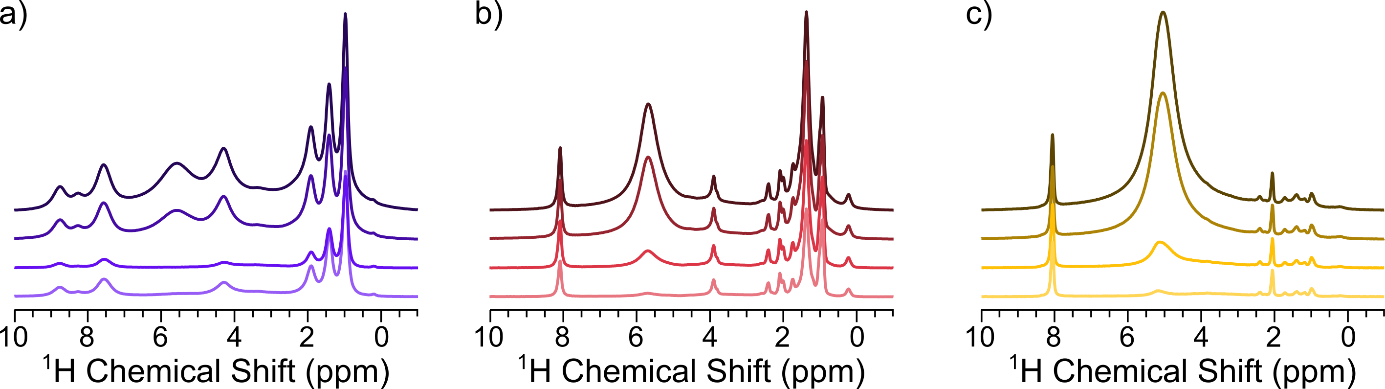


**Figure S8.** Examples of ^1^H solid-state NMR spin-echo MAS spectra of impregnated (a) Ru@SILP_IM_, (b) Ru@Si-Dec, and (c) Ru@SiO_2_, recorded with different echo times (1.0 μs, 0.2 ms, 1.5 ms and 3.0 ms from top to bottom). Longer echo delay results in lower intensities. Fitting the echo decay results in the curves shown in Figure 3. The measurements were performed at 16.4 T and 17.0 kHz MAS.


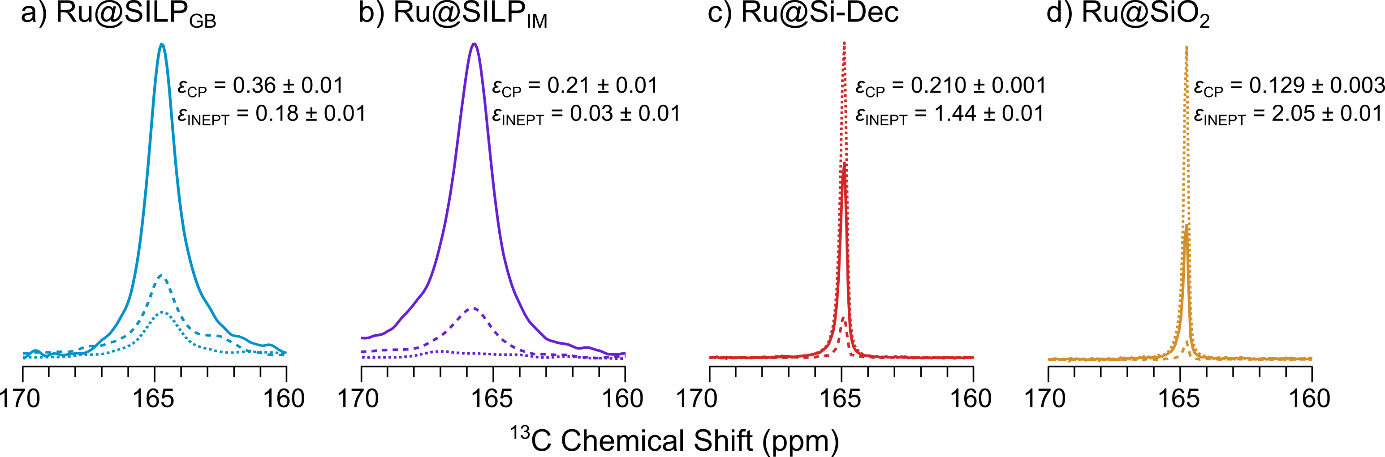


**Figure S9.** ^13^C MAS NMR spectra of the SILP materials impregnated with ^13^C-labelled formic acid, showing the region between 160 to 170 ppm, measured with DP (solid line), CP (dashed line), and INEPT (dotted line) techniques. Spectra of the same material have been normalized according to the number of scans to make the intensities directly comparable. CP and INEPT polarization transfer efficiencies (*ε*) are determined from the ratios between integrals of the CP or INEPT signal and the DP signal. All spectra were recorded at 16.4 T and a MAS frequency of 17.0 kHz.


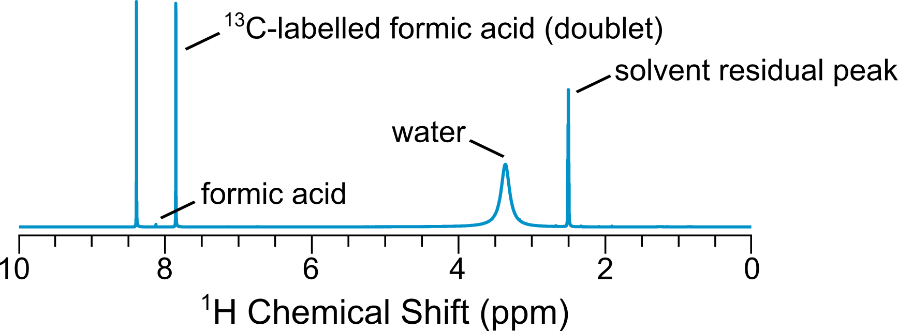


**Figure S10.** ^1^H liquid-state NMR spectrum of ^13^C-labelled formic acid in DMSO-d^6^.


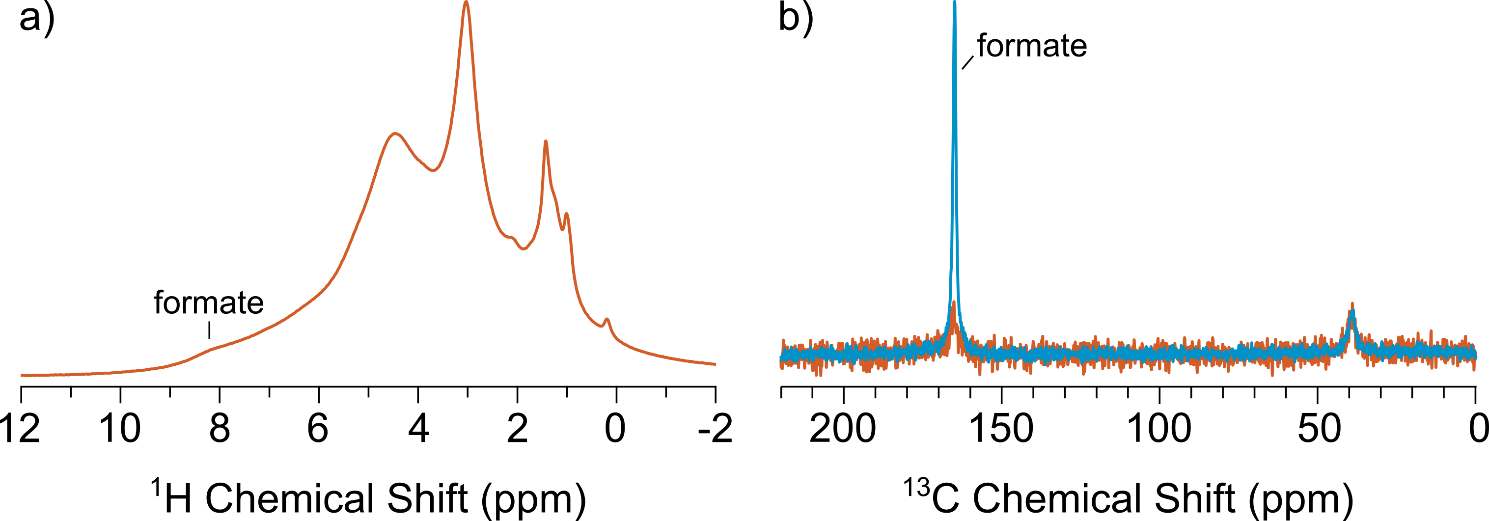


**Figure S11.** Comparison of the spectra of Ru@SILP_GB_ impregnated by ^13^C-labelled formic acid immediately after impregnation (blue) and one night later (orange). (a) ^1^H solid-state NMR spin echo spectra; (b) ^1^H-^13^C solid-state CP-MAS NMR spectra. All spectra were recorded at 16.4 T and 12.0 kHz MAS.


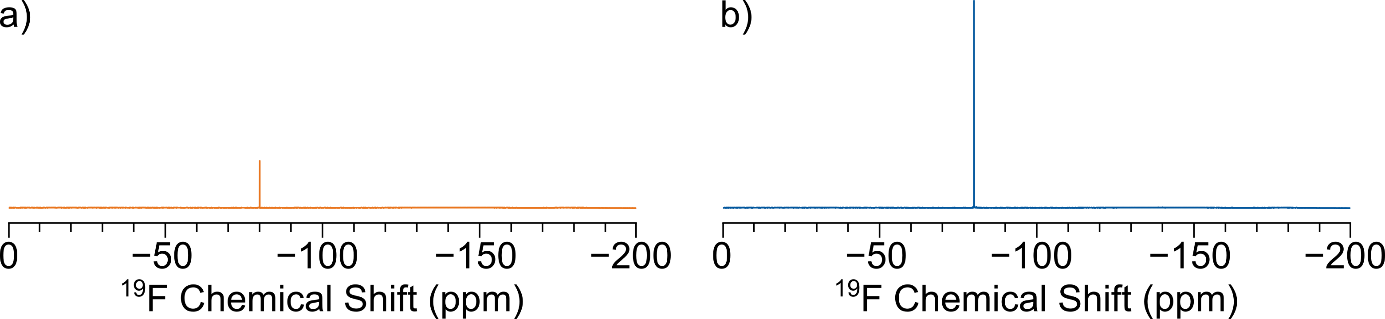


**Figure S12.** ^19^F solution-state NMR spectra of the eluent after washing SILP_GB_ with (a) ethanol and (b) 2% formic acid solution in ethanol.


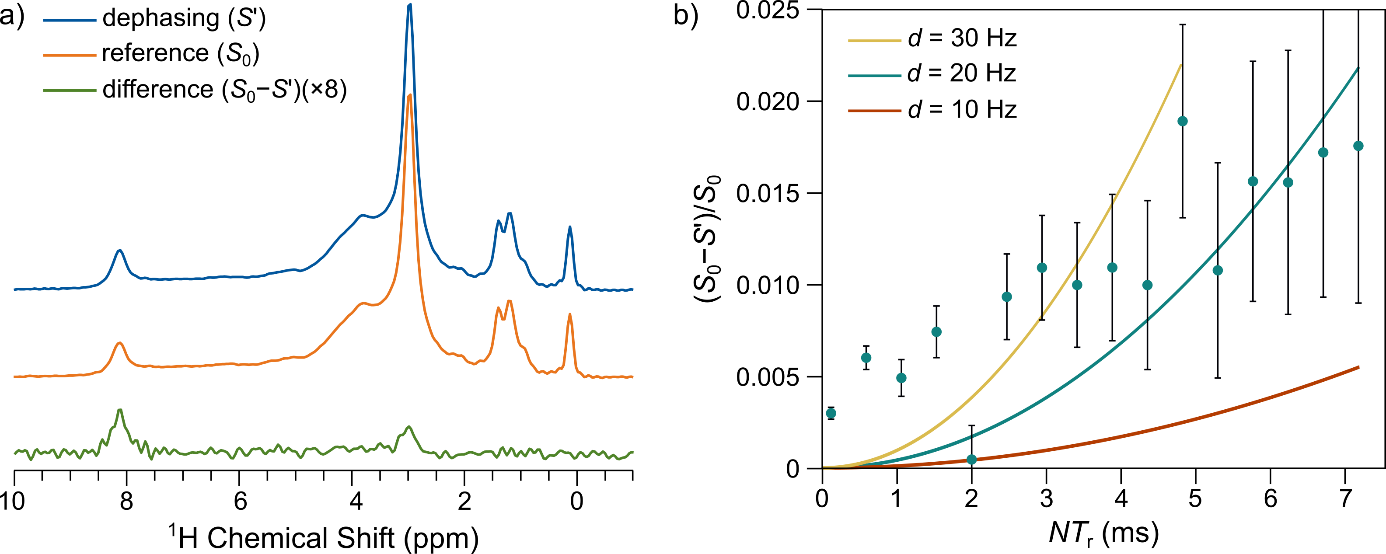


**Figure S13.** ^1^H{^13^C} REDOR experiment of Ru@SILP_GB_ impregnated with ^13^C-labelled formic acid. (a) A slice of the ^1^H{^13^C} REDOR experiment at a dephasing time of *NT*_r_ = 4.4 ms, recorded at 16.4 T and 17.0 kHz MAS. (b) REDOR dephasing as a function of different dephasing times. Straight lines show the calculated REDOR curves using the analytical solution^[7]^ assuming different nuclear dipolar coupling strengths (*d*).

Fitting Spin echo decay curves to extract *T*_2_ relaxation times

To extract the *T*_2_ relaxation times, the decay of the formate signal with increasing echo delay times was fitted to both a mono-exponential function *f*(*t*) = *a* + *b*⋅exp(-*t*/*T*_2_) and a bi-exponential function *f*(*t*) = *a* + *b*_1_⋅exp(-*t*/*T*_2,1_) + *b*_2_⋅exp(-*t*/*T*_2,2_) (Figure 3b). The results of least-square fitting are shown in Table S1-2. The bi-exponential fitting enables the separation of the contribution from the overlapping resonance of labile ^1^H, which results in a shorter relaxation time. The longer relaxation time is attributed to formate. In addition, the situation of Ru@SILP_IM_ is special because of another spectral overlap between formate and the imidazolium group on the ionic liquid (Figure S1a). Nevertheless, we noticed that ^1^H on the imidazolium relaxes at a similar rate compared to formate and a bi-exponential fitting still describes the decay curve quite accurately. Therefore, we assume the slower component from the bi-exponential fitting describes both, the formate and the imidazolium group.

**Table S1.** Results of mono-exponential fitting of spin-echo decay curves to extract *T*_2_ relaxation times.

| Material | Mono-exponential fitting | | |
| --- | --- | --- | --- |
|  | *a* | *b* | *T*_2_ (ms) |
| Ru@SILP_GB_ | 0.03 ± 0.02 | 0.94 ± 0.03 | 0.67 ± 0.07 |
| Ru@SILP_IM_ | 0.02 ± 0.01 | 0.94 ± 0.02 | 0.97 ± 0.07 |
| Ru@Si-Dec | 0.04 ± 0.03 | 0.91 ± 0.03 | 3.2 ± 0.3 |
| Ru@SiO_2_ | 0.12 ± 0.05 | 0.82 ± 0.06 | 3.2 ± 0.5 |

**Table S2.** Results of bi-exponential fitting of spin-echo decay curves to extract *T*_2_ relaxation times.

| Material | Bi-exponential fitting | | | | |
| --- | --- | --- | --- | --- | --- |
|  | *a* | *b*_1_ | *b*_2_ | *T*_2,1_ (ms) | *T*_2,2_ (ms) |
| Ru@SILP_GB_ | 0.00 ± 0.02 | 0.3 ± 0.2 | 0.7 ± 0.2 | 2 ± 1 | 0.4 ± 0.1 |
| Ru@SILP_IM_ | 0.002 ± 0.008 | 0.6 ± 0.1 | 0.4 ± 0.1 | 1.7 ± 0.3 | 0.4 ± 0.1 |
| Ru@Si-Dec | 0.01 ± 0.01 | 0.71 ± 0.03 | 0.29 ± 0.04 | 5.7 ± 0.6 | 0.5 ± 0.1 |
| Ru@SiO_2_ | 0.01 ± 0.02 | 0.61 ± 0.03 | 0.39 ± 0.02 | 11 ± 1 | 0.5 ± 0.1 |

Hydrolysis of SILP

When formic acid (1:9 v/v to CDCl_3_) was impregnated on SILP materials without Ru NPs to exclude influences of the decomposition of formate, we observed changes in the ^1^H spin echo NMR spectrum after several hours (Figure S14a). The original formate resonance at around 8.3 ppm decreased in intensity and a new resonance (around 8.2 ppm) appeared over time. We also found similar changes in the region around 4 ppm where we expect CH_2_ of the ethoxy groups on incompletely grafted ionic liquids, and for the peak at 1.4 ppm which belongs to CH_3_ of the ethoxy groups. In addition, the chemical-shift value of the labile ^1^H also significantly shifted from 6.5 ppm to 6.2 ppm. All these changes point to a depletion of the original formate and generation of some new species with the ethoxy groups whose assignment is reported below. The same changes happen faster when formic acid was used for impregnation without the assist of CDCl_3_ – which is likely leading to a poor distribution of formate on the surface and a much higher local concentration in certain regions of the sample than what was intended. The ^1^H and ^13^C resonances from formate and the new species in this case can be detected as sharp cross-peaks in a ^1^H-^13^C 2D *J* coupling-based INEPT spectrum (Figure S14b).

In order to assign these new resonances, we extracted the mobile components from the surface of the catalyst by washing the impregnated materials with deuterated dimethyl sulfoxide and recorded solution-state NMR spectra for that material (Figure S14c-f). In contrast to the extraction from SILP_GB_ not impregnated with formic acid, we found additional ^1^H and ^13^C resonances in the solid-state NMR spectra at chemical-shift values close to the formate resonances. The analysis of the ^1^H-^13^C HMBC spectrum and *J*-splitting patterns in the ^1^H spectrum suggests that the new resonances belong to an ethyl formate ester, which is formed from hydrolysis of the ethoxy groups on incompletely grafted SILPs by formic acid.


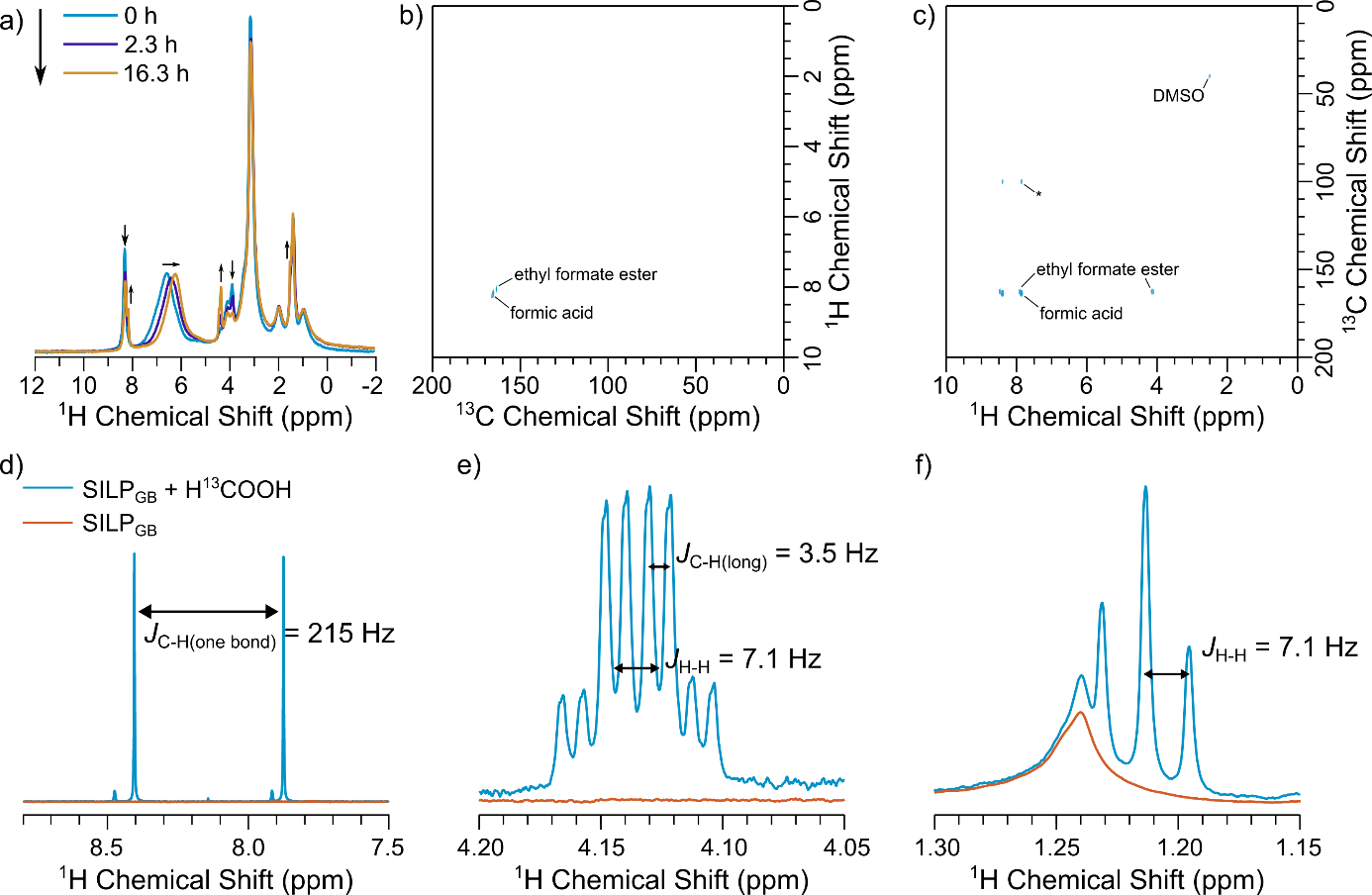


**Figure S14.** Detection of the hydrolysis of SILP. (a) ^1^H solid-state NMR spin-echo spectrum of SILP_GB_ impregnated with ^13^C-labelled formic acid at different times after preparing the sample, recorded at 16.4 T and 60.0 kHz MAS. (b) ^1^H-^13^C 2D solid-state *J*-coupling-based INEPT spectrum of SILP_GB_ impregnated by ^13^C-labelled formic acid without CDCl_3_, recorded at 16.4 T and 17.0 kHz MAS. (c) ^1^H-^13^C HMBC spectrum of the dimethyl sulfoxide extraction. (d-f) Parts of the ^1^H solution-state NMR spectrum of the dimethyl sulfoxide solution after washing the original SILP_GB_ and SILP_GB_ impregnated with ^13^C-labelled formic acid.

**Supplementary references**

[1] Y. Zhang, N. Levin, L. Kang, F. Müller, M. Zobel, S. DeBeer, W. Leitner, A. Bordet, *J. Am. Chem. Soc.* **2024**, *146*, 30057–30067.

[2] R. Harris, E. Becker, C. de Menezes, R. Goodfellow, P. Granger, *Pure Appl. Chem.* **2001**, *73*, 1795–1818.

[3] W. L. Earl, D. L. Vanderhart, *J. Magn. Reson.* **1982**, *48*, 35–54.

[4] A. J. Shaka, J. Keeler, T. Frenkiel, R. Freeman, *J. Magn. Reson.* **1983**, *52*, 335–338.

[5] S. Hediger, B. H. Meier, N. D. Kurur, G. Bodenhausen, R. R. Ernst, *Chem. Phys. Lett.* **1994**, *223*, 283–288.

[6] S. Hediger, B. H. Meier, R. R. Ernst, *Chem. Phys. Lett.* **1995**, *240*, 449–456.

[7] K. T. Mueller, *J. Magn. Reson., Ser. A* **1995**, *113*, 81–93.

[8] A. G. M. Rankin, P. B. Webb, D. M. Dawson, J. Viger-Gravel, B. J. Walder, L. Emsley, S. E. Ashbrook, *J. Phys. Chem. C* **2017**, *121*, 22977–22984.

[9] B. M. Fung, A. K. Khitrin, K. Ermolaev, *J. Magn. Reson.* **2000**, *142*, 97–101.

[10] H. E. Gottlieb, V. Kotlyar, A. Nudelman, *J. Org. Chem.* **1997**, *62*, 7512–7515.

[11] D. Massiot, F. Fayon, M. Capron, I. King, S. Le Calvé, B. Alonso, J.-O. Durand, B. Bujoli, Z. Gan, G. Hoatson, *Magn. Reson. Chem.* **2002**, *40*, 70–76.

[12] W. F. Vranken, W. Boucher, T. J. Stevens, R. H. Fogh, A. Pajon, M. Llinas, E. L. Ulrich, J. L. Markley, J. Ionides, E. D. Laue, *Proteins:Struct., Funct., Bioinf.* **2005**, *59*, 687–696.
